# Insights on the Relationship Between Hippocampal Connectivity and Memory Performances at the Early Stage of Multiple Sclerosis

**DOI:** 10.3389/fneur.2021.667531

**Published:** 2021-05-19

**Authors:** Juliette Boscheron, Aurélie Ruet, Mathilde Deloire, Julie Charré-Morin, Aurore Saubusse, Bruno Brochet, Thomas Tourdias, Ismail Koubiyr

**Affiliations:** ^1^Univ. Bordeaux, INSERM, Neurocentre Magendie, U1215, Bordeaux, France; ^2^CHU de Bordeaux, Service de Neurologie, Bordeaux, France; ^3^CHU de Bordeaux, Neuroimagerie diagnostique et thérapeutique, Bordeaux, France

**Keywords:** multiple sclerosis, clinically isolated syndrome, memory, hippocampus, functional connectivity, structural connectivity, graph theory

## Abstract

While memory impairment in multiple sclerosis (MS) is known to be associated with hippocampal alterations, whether hippocampal networks could dynamically reorganize as a compensation mechanism is still a matter of debate. In this context, our aim was to identify the patterns of structural and functional connectivity between the hippocampus and the rest of the brain and their possible relevance to memory performances in early MS. Thirty-two patients with a first episode suggestive of MS together with 10 matched healthy controls were prospectively explored at baseline, 1 and 5 years follow up. They were scanned with MRI and underwent a neuropsychological battery of tests that included the Selective Reminding Test and the Brief Visual Memory Test Revised to assess verbal and visuo-spatial memory, respectively. Hippocampal volume was computed together with four graph theory metrics to study the structural and functional connectivity of both hippocampi with the rest of the brain. Associations between network parameters and memory performances were assessed using linear mixed-effects (LME) models. Considering cognitive abilities, verbal memory performances of patients decreased over time while visuo-spatial memory performances were maintained. In parallel, hippocampal volumes decreased significantly while structural and functional connectivity metrics were modified, with an increase in hippocampal connections over time. More precisely, these modifications were indicating a reinforcement of hippocampal short-distance connections. LME models revealed that the drop in verbal memory performances was associated with hippocampal volume loss, while the preservation of visuo-spatial memory performances was linked to decreased hippocampal functional shortest path length. In conclusion, we demonstrated a differential impairment in memory performances in the early stages of MS and an important interplay between hippocampal-related structural and functional networks and those performances. As the structural damage increases, functional reorganization seems to be able to maintain visuo-spatial memory performances with strengthened short-distance connections.

## Introduction

Multiple sclerosis (MS) is a chronic, inflammatory, demyelinating, and neurodegenerative disorder of the central nervous system. The progression of the disease is typically characterized by physical disability such as motor or sensory symptoms that are related to the recurrence of inflammatory attacks. In addition to those symptoms, 40–70% of MS patients also experience cognitive impairments (1) which can appear early in the course of the disease, even at the stage of clinically isolated syndrome (CIS), the first episode suggestive of further MS. It is now accepted that cognitive impairment in MS is negatively associated with quality of life and strongly impacts vocational status and rate of unemployment (2).

Different cognitive domains can be impaired in the context of MS such as memory, information processing speed or executive functions, with some inter-patient variability (1). Amongst these different domains, memory is one of the most consistently impaired with approximately half of the patients concerned (3). Nevertheless, the pathophysiology of memory impairment in MS is still a matter of debate and should be clarified in order to target therapeutic strategies including specific cognitive rehabilitation programs.

Most studies now agree on hippocampal involvement. Post-mortem pathological studies and *in vivo* MRI studies have pointed toward a vulnerability of the hippocampus to the inflammatory environment associated with MS. Indeed, post-mortem studies of MS patients have reported hippocampal demyelination, neural loss, and a decreased expression of neuronal proteins, ultimately leading to tissue atrophy (4, 5). In addition, *in vivo* MRI studies have also been able to capture such structural damages in terms of hippocampal volume loss, alteration of microstructural metrics or modification of structural connectivity: all of them showing some degree of correlation with memory impairment in MS patients (6–10).

However, whether functional reorganization could help compensate such damages to mitigate memory deficit is a matter of intense debate. Indeed, functional MRI (fMRI) can now be used to explore non-invasively the functional activity of the brain during a task or at rest, in the so-called resting state fMRI (rs-fMRI). From rs-fMRI, Schoonheim et al., proposed that a compensatory mechanism could be put into play in the form of a functional reorganization of networks to compensate for structural alterations induced by the disease and to mitigate clinical deficits (11). This theory would explain a delay in cognitive impairment appearance after the onset of the disease and is important because, if true, it could justify the “stimulation” of such alternate networks through specific rehabilitation and training programs. However, different reports provided conflicting data with respect to this model. Some authors have reported a decrease in functional connectivity in memory impaired compared to preserved MS patients which could be interpreted as a lack of compensation (6, 12). Comparable results were reported in an activation study where cognitively preserved patients showed an increase in activation of hippocampal memory system compared to healthy controls when performing a memorization task, while cognitively impaired patients showed less activation (13). Differently, an increase in functional connectivity among core part of the default mode network (6, 14) and between the right hippocampus and frontal areas (7) was associated with loss of cognitive efficiency rather than with preserved functions. Several limitations could explain such conflicting results; the most important being the cross-sectional designs of all these studies, with MS patients at different stages of the disease and without joined analyses of structural and functional metrics.

In this context, our aim was to identify the patterns of structural and functional connectivity between the hippocampus and the rest of the brain and their possible relevance to maintain memory performances in MS. We hypothesized that functional reorganization could compensate for structural damage, allowing a delay in memory impairment appearance.

To explore this question, we used a multimodal approach, combining *in vivo* structural measures—i.e., hippocampal volume and structural connectivity—and functional measures—i.e., rs-fMRI connectivity. We took advantage of a prospective longitudinal cohort of patients—and matched healthy controls—explored at the early stage (CIS), and followed at 1 and 5 years with an extensive MRI protocol and a large neuropsychological battery including tests to assess verbal and visual memory. This longitudinal setting from the beginning of the disease was unique to observe how memory impairment evolves during the pathology course and how the structural and functional connectivity of the hippocampus are linked to this evolution.

## Materials and Methods

### Population

A prospective cohort of 32 patients who experienced a first episode suggestive of MS was recruited, <6 months after the episode. All participants provided an informed written consent, and an ethical committee approved the study (SCI-COG, ClinicalTrials.gov Identifier: NCT01865357). Inclusion criterion was to present with at least two clinically silent cerebral lesions characteristic of MS on fast fluid-attenuated inversion recovery (FLAIR) images. As for exclusion criteria, they included age below 18 years, inability to undergo MRI, history of other neurological or psychiatric disorders, MS relapse within 2 months prior to screening, corticosteroid pulse therapy within 2 months prior to screening, and severe depression [Beck Depression Inventory (15) >27]. Ten healthy controls matched for age, sex, and educational level were also included. All MS patients and healthy controls underwent a neuropsychological assessment at baseline as well as at a year 1 and year 5 follow-up. The Expanded Disability Status Scale (EDSS) scores were determined for patients at the three time points by expert neurologists and conversion (or not) to MS was judged according to 2017 McDonald criteria (16). Patients also underwent an MRI scan at the three time points, while healthy controls were only scanned at baseline and at the 5-year follow-up.

### Neuropsychological Assessment

Episodic memory efficiency was assessed by two different tests: the Selective Reminding Test (SRT) (17), to evaluate episodic verbal memory performances (three sub-scores: SRT-LTS = long-term storage; SRT-CLTR = consistent long-term retrieval; SRT-DR = delay recall) and the Brief Visual Memory Test Revised (BVMT-R) (18), to evaluate episodic visuospatial memory performances (two sub-scores: BVMTR = learning; BVMTR-DR = delayed recall). All participants also underwent a comprehensive neuropsychological battery of tests. In order to account for practice effects (test-retest effect), we compared patients' scores with healthy controls' scores at each time point (baseline, 1- and 5-year follow-up) by using *Z*-scores.

### MRI Acquisition

Imaging was performed using 3 Tesla MRI systems (Achieva TX system, Philips Healthcare, Best, The Netherlands; Signa, GE Healthcare, Discovery MR 750w, Milwaukee, Wisconsin). Structural images were acquired with a 3D T1-weighted sequence using magnetization prepared rapid gradient echo (MP-RAGE) imaging (TR = 8.2 ms, TE = 3.5 ms, TI = 982 ms, α = 7°, FOV = 256 mm, voxel size = 1 mm^3^, and 180 slices) as well as a 2D FLAIR sequence (TR = 11,000 ms, TE = 140 ms, TI = 2,800 ms, FOV = 230 mm, 45 axial slices, and 3-mm thick). Diffusion images were acquired with a diffusion tensor echo-planar-imaging pulse sequence (TR = 11,676 ms, TE = 60 ms, FOV = 230 mm, voxel size = 1.6 mm^3^) in 21 non-colinear directions at b = 1,000 s/mm^2^, and with one b = 0 s/mm^2^. Finally, resting-state functional images were acquired with a whole-brain T2^*^-weighted echo-planar imaging (EPI) sequence (250 volumes, 40 axial slices, TR = 2,200 ms, TE = 30 ms, voxel size = 3 mm^3^). The first four volumes of the functional run were removed to reach signal stability.

### Structural Preprocessing and Parcellation

The Lesion Segmentation Tool (LST) version 2.0.15 of SPM12 (http://www.applied-statistics.de/lst.html) was used to segment MS lesions on FLAIR data. Lesions were further manually corrected by two blinded experts. In order to prevent brain tissue segmentation from being biased by lesions, those masks of segmented lesions were used to apply a lesion-filling algorithm to the T1-weighted images. Whole-brain, total white-matter, gray-matter and hippocampal volumes were calculated using the volBrain system (https://volbrain.upv.es/). The segmentation procedure consists first of denoising and inhomogeneity correction, after which volumes are affine registered to the Montreal Neurological Institute (MNI) space. To control for variations in head size, each volume was assessed as a fraction of total intracranial volume (TIV). Subsequently, FreeSurfer (v5.3) image analysis suite (http://surfer.nmr.mgh.harvard.edu) was used to preprocess structural data and separate them into parcels using a custom-made atlas based on Destrieux cortical atlas (19). The latter consists of a parcellation originating from the division of the neocortex into gyral and sulcal regions, both being delineated by the curvature value of the surface. In addition, deep gray matter structures (i.e., pallidus, accumbens, putamen, caudate, and amygdala), the cerebellar cortex and the ventral diencephalon, were also included as parcels. At the end, we obtained a custom-made atlas which included 83 parcels per hemisphere. This parcellation was used to compute the structural connectivity between both hippocampi and each individual parcel (see below).

### DTI Preprocessing

Diffusion data were preprocessed using the Oxford Center for Functional MRI of the Brain (FMRIB) Software Library (FSL, version 5.0.9; fsl.fmrib.ox.ac.uk/fsl) and MRtrix3 software (20) was used for diffusion-weighted tractography. We first corrected for motion artifacts and eddy current distortions. Next, fiber orientation distributions were calculated using the constrained spherical-deconvolution algorithm (21). About 10 million whole-brain streamlines were subsequently generated using the five-tissue-type segmented T1 image and the anatomically constrained tractography (20). These streamlines were cropped at the gray matter–white matter interface and further filtered to about 2 million using the spherical-deconvolution informed filtering of tractograms (22) to reduce reconstruction bias and improve biological plausibility. Finally, T1-weighted images were registered to diffusion images (b0 image as a reference) by a rigid registration followed by a non-rigid registration of the T1-weighted image to the subject's b0 space using ANTs software (23). Following this registration, the previously obtained streamlines were mapped into the 166 nodes (83 per hemisphere) of the custom-made atlas and a structural connectivity 166 × 166 matrix was computed. Each element of the matrix represents the number of streamlines between two regions normalized by the total number of streamlines for each participant, accounting for region size. Structural connectome matrices for patients are displayed in Supplementary Figure 1.

### fMRI Preprocessing

fMRI pre-processing of images was performed using publicly available software (SPM12, FSL) following the same procedure as the one used by Yeo et al. (24). The 4 first scans of all participants were removed to reach signal stability. First, slice acquisition-dependent time shifts between volumes were compensated for. Second, head motion was corrected using rigid body translation and rotation and 6 parameters were extracted. Next, constant offset and linear trend over each run were removed and a low-pass filter was applied (0.08 Hz). Finally, the whole brain mean signal, the mean signal within the white matter and the mean signal within the ventricles were regressed out, together with the 6 motion parameters extracted during the previous step and their temporal derivatives. This last regression step aims at minimizing non-neuronal signal contributions, such as respiration-induced signal fluctuations. fMRI sequences were registered to the 3D T1 sequences with a boundary-based procedure and further visually checked. In order to analyze the blood-oxygen level dependent (BOLD) signal of the pre-processed volumes, a region-based approach was chosen. The parcellation is detailed in “structural preprocessing” section. For each parcel, the average of the BOLD time course signal of voxels belonging to this parcel was computed. Pairwise Pearson correlations between the BOLD signal of each region with all the remaining 165 regions were computed, resulting in a 166 × 166 functional connectivity matrix for each subject. Finally, a Fisher's *Z*-transformation was applied to the correlation matrices to improve normality. Functional connectome matrices for patients are displayed in Supplementary Figure 2.

### Connectivity Metrics

Network analysis was performed using the Brain Connectivity Toolbox (http://www.brain-connectivity-toolbox.net) (25). In order to study the structural and functional connectivity of both hippocampi with all other brain regions we focused on four metrics, coming from graph theory: strength and betweenness centrality to represent centrality properties, the average shortest path length (SPL) showing integration properties, and the clustering coefficient representing segregation properties. The strength of a node (e.g., the right or left hippocampus) is the sum of all connections it possesses with the rest of the brain. The average SPL of a node is the mean of all shortest paths between this node and all the others. The shortest path between two nodes is defined as the inverse of the sum of all connections constituting the shortest path between the two nodes. The betweenness centrality of a node is the fraction of all shortest paths in the network (i.e., the whole brain) that contain this node. Nodes with high values of betweenness centrality participate in a large number of shortest paths and thus represent the core of the network. The clustering coefficient is the fraction of a node's neighbors that are neighbors of each other. Nodes with high values of clustering coefficient are surrounded by other nodes which altogether form a cluster. We computed a mean of both right and left hippocampi for each connectivity metric.

### Statistical Analysis

Statistical analyses were performed using R software (version 3.4.2, https://www.r-project.org) and SPSS 23.0 (SPSS, Chicago, IL, USA). Shapiro–Wilk test was used to test for normality of distribution. Depending on the distribution of our variables either parametric or non-parametric tests were used.

The evolution of cognitive variables over time was evaluated using paired Student *t*-tests and Wilcoxon tests depending on the distribution of each variable and significant *p*-values were extracted after Bonferroni's correction for multiple comparison.

For the evolution of MRI variables, in order to take into account possible confounding factors, we analyzed age-, sex-, education-, and scanner-standardized residuals of MRI metrics which were compared between baseline and year 5 in controls, and at each of the three time periods (baseline/year 1, baseline/year 5, and year 1/year 5) in patients. Paired Student *t*-tests and Wilcoxon tests were used depending on the distribution of residuals and significant *p*-values were extracted after Bonferroni's correction for multiple comparison.

To evaluate the link between patients' scores in memory tests and MRI metrics over time (i.e., baseline, year 1 and year 5 follow-up), we fitted linear mixed effects (LME) models with a random intercept term calculated for each patient. For each sub-score of the two memory tests, we fitted four LME models (one for each MRI metric significantly altered over time). Cognitive *z*-scores were the dependent variables and age-, sex-, education-, and scanner-standardized residuals of altered network measures were the predictor variables. The predictive power of each model was assessed using the Bayesian information criterion (BIC). The estimate of each random effect was further extracted together with the associated *p*-value after Bonferroni's correction for multiple comparison.

## Results

In this study we observed how memory performances evolve in the course of MS, since its onset, and how hippocampal volume together with hippocampal structural and functional connectivity can be linked to this evolution.

### Patients Demographic, Clinical and Conventional MRI Characteristics

This study included 32 patients and 10 healthy controls whose characteristics were matched.

EDSS scores did not change significantly between baseline and year 1 (*p* = 0.798), nor between year 1 and year 5 (*p* = 0.086) but did increase significantly between baseline and year 5 (*p* < 0.05) (Table 1). T2 lesion volumes, on the other hand, did not differ significantly between baseline and year 1 (*p* = 0.784), nor between baseline and year 5 (*p* = 0.065), but did increase significantly between year 1 and year 5 (*p* < 0.001) (Table 1). Interestingly, whole-brain volume significantly decreased 1 year after the disease onset (*p* < 0.05). We found that this was mainly driven by alterations of white matter whose mean volume significantly decreased at the 1-year (*p* < 0.01) and at the 5-year (*p* < 0.05) follow-up while gray matter did not change significantly.

**Table 1:** Patients demographic, clinical and conventional MRI characteristics.

	**Baseline**		**1 year**	**5 years**
**Population**	**Patients**	**Controls**	**Patients**	**Patients**
Mean age, years *(SD)*	37.8 (10.4)	40.4 (7.06)	–	–
Sex ratio *(F/M)*	25/7	6/4	–	–
Education level *(high/low)*^a^	20/12	9/10	–	–
Median EDSS score *[range]*^b^	1.5 [0–3]	–	1 [0–3]	1.75 [0–4]^#^
Median T2 Lesion volume *mL [range]*^b^	0.85 [0.02–25.97]	–	1.56 [0.07–16.65]	2.40 [0.17–20.97]
Conversion to MS *n (%)*	28 (87.5)	–	29 (90.6)	29 (90.6)
Normalized brain fraction % (SD)^c^	84.67 (3.43)	85.43 (2.52)	83.95 (3.72)^*^	84.13 (4.27)
Normalized white matter fraction % (SD)^c^	35.60 (2.83)	37.00 (2.70)	34.44 (3.21)^**^	34.62 (2.71)^#^
Normalized gray matter fraction % (SD)^c^	49.07 (2.95)	48.42 (1.79)	49.51 (2.85)	49.51 (3.20)

*SD, standard deviation; EDSS, Expanded Disability Status Scale*.

a *French baccalaureate/no French baccalaureate;*

b *Wilcoxon test;*

c *Paired t-test. Comparison between baseline and 1-year follow-up:*

* *p < 0.05;*

** *p < 0.01. Comparison between 1- and 5-year follow-up: p < 0.001. Comparison between baseline and 5-year follow-up:*

# *p < 0.05*.

### Memory Performances of Patients at Baseline, Year 1 and Year 5

**Figure 1:**
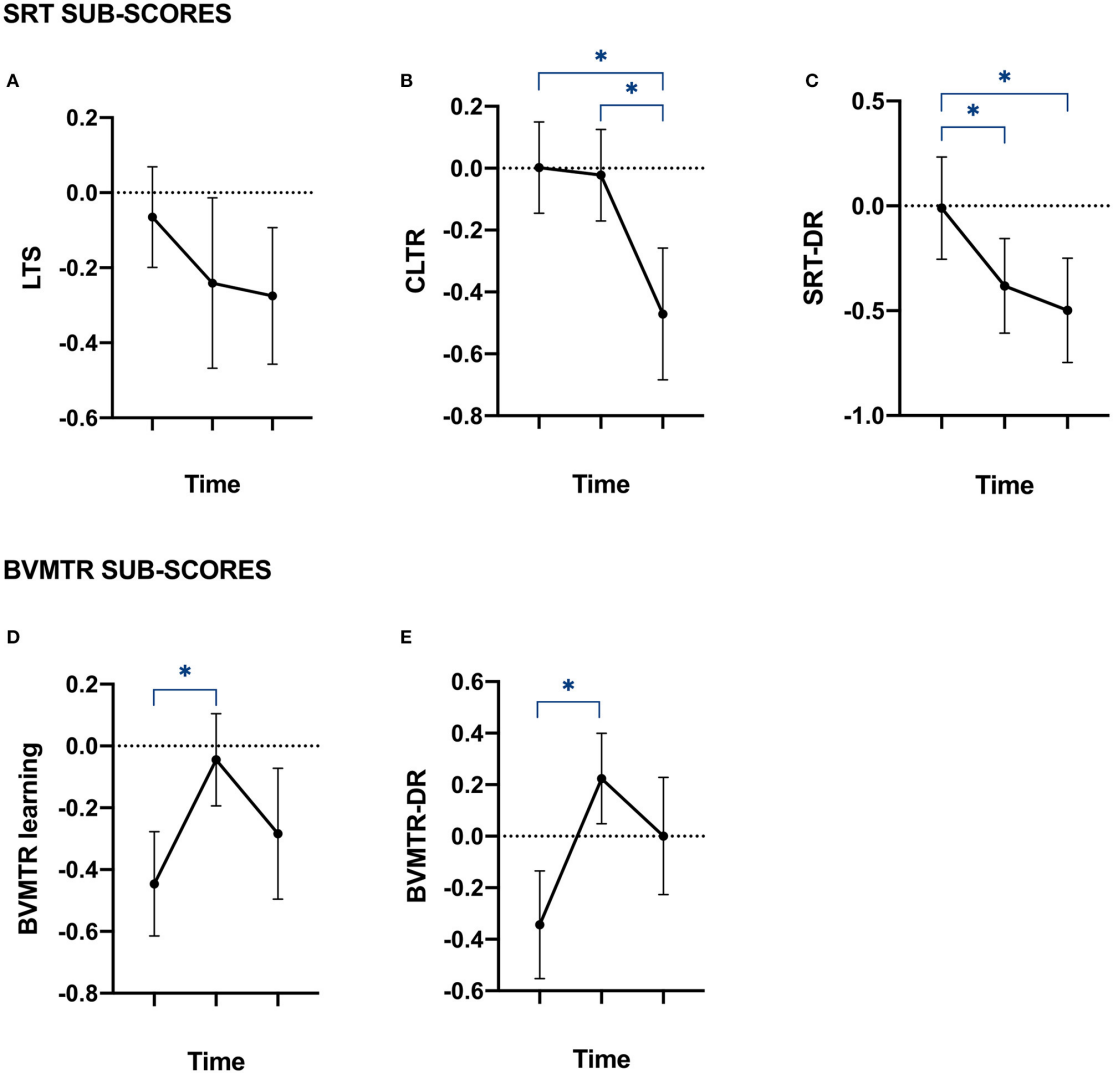
Memory performances of patients at baseline, year 1 and year 5. Plots of patients' *Z*-scores to the tests assessing episodic memory. Data are provided as mean with standard error of the mean. **(A,B)** plots represent the *Z*-scores of patients on each of the Selective Reminding Test (SRT) sub-items, assessing episodic verbal memory performances. **(A)** LTS, long-term storage; **(B)** CLTR, consistent long-term retrieval; and **(C)** SRT-DR = delay recall. **(D,E)** plots represent the *Z*-scores of patients on each of the Brief Visual Memory Test Revised (BVMTR) sub-items, assessing episodic visuospatial memory performances. **(D)** BVMTR = learning; and **(E)** BVMTR-DR = delayed recall. *Correspond to significant *p*-value after Bonferroni's correction for multiple comparison.

Figure 1 reports patients *z*-scores to each sub-item of the two memory tests performed (SRT and BVMTR). Verbal and visuospatial memory performances were differentially affected in patients over time.

The LST was the only sub-item of both tests which did not show a significant impairment over time (Figure 1A). Indeed, the CLTR sub-item of the SRT decreased significantly between baseline and year 5 as well as between year 1 and year 5 (Figure 1B). This was observed together with a significant decrease of the SRT-DR sub-item of the SRT between baseline and year 1 and between baseline and year 5 (Figure 1C). Additionally, a significant increase of both BVMTR sub-items was observed between baseline and year 1 (Figures 1D,E).

Raw scores of patients to each cognitive test can be found in Supplementary Table 1.

Those results suggest that patients do not display the same learning-effect in verbal memory as it is seen in healthy controls after 5 years of evolution, i.e., patients learn less. Visuo-spatial memory on the other hand, seems to be maintained.

### Hippocampal Volume and Connectivity at Baseline, Year 1 and Year 5

**Figure 2:**
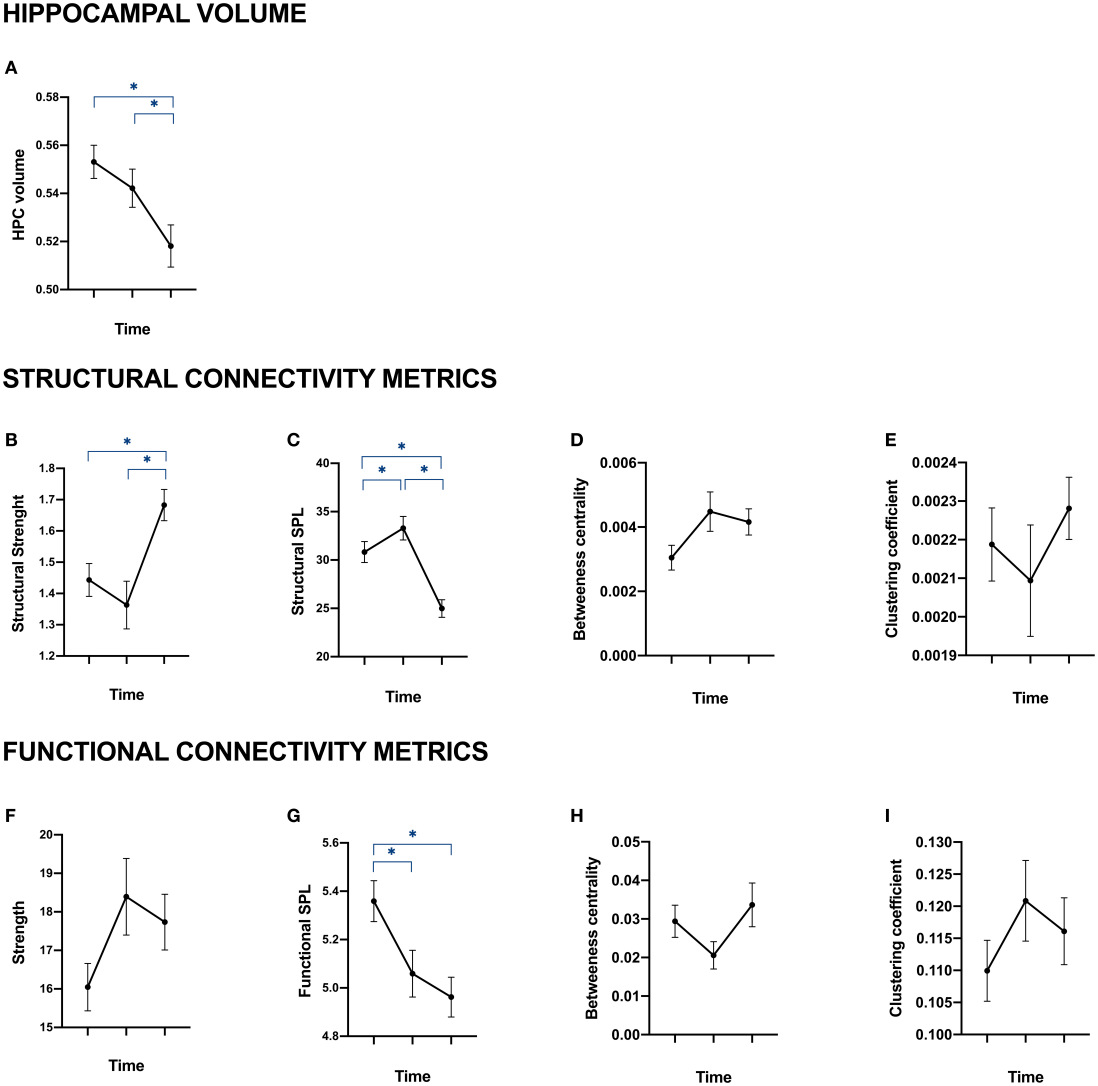
Hippocampal volume, structural and functional connectivity of patients at baseline, year 1 and year 5. Data are provided as mean with standard error of the mean. **(A)** represents the evolution over time of patients' total hippocampal volume (sum of right and left hippocampi). **(B–E)** represent the mean of right and left hippocampi structural connectivity through four metrics coming from graph theory: strength **(B)**, average shortest path length **(C)**, betweenness centrality **(D)** and clustering coefficient. **(F–I)** represent hippocampal functional connectivity through the same metrics: strength **(F)**, average shortest path length **(G)**, betweenness centrality **(H)** and clustering coefficient **(I)**. *Correspond to significant *p*-value after Bonferroni's correction for multiple comparison on age-, sex, education-, and scanner-standardized residuals.

Figure 2 shows the evolution of MRI metrics over the three time points in patients. A significant decrease in hippocampal volumes was observed from baseline to year 5 and from year 1 to year 5 (Figure 2A). This was present along with a significant increase in the structural strength of connections between hippocampi and the rest of the brain over the same periods of time (Figure 2B). Functional strength, on the other hand, was not significantly altered over time (Figure 2F). Additionally, we observed a significant decrease of structural and functional SPL between hippocampi and the rest of the brain when comparing baseline and year 1 and baseline and year 5 (Figures 2C,G). As for betweenness centrality and clustering coefficient, they were not significantly altered in structural (Figures 2D,E) nor functional (Figures 2H,I) hippocampal networks.

As for healthy controls, none of the standardized residuals of hippocampal structural and functional connectivity metrics were significantly altered over time (data not shown).

### Link Between Memory Performances and MRI Metrics at Baseline, Year 1 and Year 5

In order to evaluate the associations between patients' scores to memory tests (see Figure 1) and MRI metrics (see Figure 2) over time, we fitted LME models.

In a first series of models, our dependent variables were *z*-scores of patients to each sub-item of the SRT (LTS, CLTR, and SRT-DR). We found that CLTR *z*-scores were significantly explained by hippocampal volume (Estimate = 0.29; *p* < 0.01; BIC = 238.49); lower scores being associated with lower hippocampal volume. A sensitivity analysis showed that this association was driven by the left hippocampus (Estimate = 0.33; *p* < 0.01; BIC = 236.65). However, there was no contribution of our connectivity metrics to the CLTR. On the other hand, LTS and SRT-DR *z*-scores were not significantly explained by any of the MRI metrics which showed an evolution over time.

In a second serie of models, we explored the sub-items of the BVMTR. BVMTR-DR *z*-scores were significantly explained by hippocampal functional SPL (Estimate = −0.33, *p* < 0.01; BIC = 288.73); better scores being associated with lower values of SPL. A sensitivity analysis showed that this association was driven by the right hippocampus (Estimate = −0.345, *p* < 0.001; BIC = 287.57). There was no contribution of the other MRI metrics to the BVMTR-DR. On the other hand, BVMTR-learning *z*-scores were not significantly explained by any of the MRI metrics which showed an evolution over time.

## Discussion

In this study we shed light on the evolution of memory performances throughout time in the context of early MS and observed their association with hippocampal structural and functional alterations. While we confirmed the already reported decrease in hippocampal volume and its association with some memory dysfunction, we provided new data regarding network reorganization compatible with phenomena of compensation. Indeed, we found data interpreted as a progressive increase in connections between both hippocampi and the rest of the brain with preference for reinforcement of short distance connections which were associated with maintained memory performances in some domains.

### Hippocampal Volume Loss and Verbal Memory Decline in MS Patients

#### Hippocampal Atrophy

We observed a significant decrease in hippocampal volume between baseline, year 1 and year 5, indicating progressive tissue alteration from the early stages of the disease. These data are in line with demyelination and neuronal loss that were reported on pathological examinations from post-mortem brain (5). This is also in line with previous *in vivo* MRI studies which have robustly reported hippocampal volume loss in MS patients compared to healthy controls (6, 7); as well as in MS patients across time (9, 26). The differential vulnerability of the hippocampus to MS pathology was confirmed in our data by the observation that no significant evolution was observed across the five-year period in whole brain gray-matter volumes. This stability in whole brain gray-matter volumes over time could be explained by the presence of multiple local atrophies—such as the one reported here in the hippocampus—which are not yet pronounced enough to be visible in the global picture. Fleischer et al. (27) reported a similar result with no significant alteration in GM volume in MS patients at different disease stages but a significant reorganization of GM networks.

#### Relation With Verbal Memory Performances

Regarding the major role of the hippocampus in memory functions (28), the overall decrease in verbal memory performances between baseline, year 1 and year 5 was not unexpected in this context of hippocampal atrophy. Accordingly, our LME models revealed that hippocampal volume was significantly associated with patients' verbal memory performances over time—i.e., it could explain the CLTR sub-item of the SRT. These data confirmed the early memory decline in the context of MS (10, 29) and the implication of specific hippocampal neurodegeneration in such a cognitive decline (9, 26).

On the other hand, patients' visuospatial memory performances were maintained over time despite the significant hippocampal atrophy that we reported. This raises the possibility that additional mechanisms of compensation could be involved, such as reorganization of hippocampal networks.

### Hippocampal Networks Reorganization and Visuo-Spatial Memory Maintenance in MS Patients

As a matter of fact, our analysis revealed an important reorganization both in terms of structural and functional hippocampal connectivity.

#### Structural Reorganization

First, we observed a significant increase of hippocampal structural strength over time in patients, denoting an increase in the number of connections linking both hippocampi to the rest of the brain. This finding is in line with a previous study reporting greater structural connectivity between both thalami in MS patients compared to healthy controls (30). Such structural plasticity was also reported in many rehabilitation studies of MS patients [see (31) for a review]. Additionally, a significant decrease of hippocampal structural SPL was observed, suggesting a greater efficacy of hippocampal networks, which could be associated to the raise in structural strength that we reported. The increase observed in hippocampal structural strength could be a response to disease pathology as it was previously observed by Fleischer et al. in 2016 (27). They hypothesize that structural reorganization occurs to compensate for ongoing diffuse damage and are essential to maintain network functioning (27). Another interpretation for this increase in detected hippocampal fibers might actually be a reorganization of hippocampal functional connectivity. Indeed, it was previously suggested that activated cells undergo biophysical changes, such as cell swelling and membrane expansion in case of active neuronal firing (32); a phenomenon which could increase the DTI-based detectability of some fibers. Therefore, the increase of hippocampal structural connectivity observed in this study could be considered as a physiological marker of neuronal activation. A qualitative analysis of hippocampal structural connectivity (Supplementary Figure 1) indicated an increase in connections between both hippocampi and left temporal regions. Although this was out of the scope of our analysis, future studies should investigate alterations of specific connections and their impact on memory performances.

#### Functional Reorganization

Indeed, a significant decrease of hippocampal functional SPL was observed, indicating a reinforcement of existing hippocampal functional connections and/or the functional synchronization of the hippocampus with new brain regions. Moreover, a drop in SPL usually characterizes an increase in local, short-distance connections (33). This is in line with a previous study in which it was shown that long-range connections were more severely damaged by multiple sclerosis pathology (34). Fleischer et al. (27) reported similar observations with a strengthening of local connections in the first year after disease onset. Finally, short-distance brain regions are known to be more densely connected both in terms of axonal projections and functional connectivity strength, due to metabolic reasons such as wiring cost (35). The fact that we do not observe a congruent increase in hippocampal functional strength could be explained by an overall equilibrium of functional reorganization. Indeed, even if some hippocampal functional connections are strengthened—mainly short-distance ones—others are weakened with the evolution of the disease (36).

#### Relation With Visuo-Spatial Memory Performances

LME models revealed that patients' scores to the delayed recall sub-item of the BVMTR were significantly explained by hippocampal functional SPL over time. We also saw that visuo-spatial memory performances—assessed by the BVMTR—were maintained throughout time in MS patients. We can thus speculate that the functional reorganization observed is compensating for hippocampal volume loss, allowing the maintenance of such performances. This hypothesis is in line with Schoonheim et al., suggesting that functional reorganization can act as a compensatory mechanism to attune for structural alterations induced by the disease and mitigate clinical deficits (11). It is also coherent with the results reported by Hulst et al. where the activity of hippocampal memory system was increased in cognitively preserved patients compared to healthy controls when encoding correctly remembered items (13). Additionally, it was previously reported that MS patients' performances in a dual-task were negatively correlated with resting-state networks modularity values; again, suggesting a link between cognitive performances and functional reorganization (37).

However, the BVMTR-learning sub-item of the BVMTR—which did not show impairment across time either—could not be significantly explained by any of our MRI metrics. This gives some perspective on our interpretation of preserved cognitive functions being associated with functional reorganization. Additionally, it is important to notice that even though no significant link was observed between hippocampal structural reorganization and the maintenance of visuo-spatial memory performances, the interplay between both might be of interest. Indeed, it was previously suggested that an increase in structural short-distance connections could be partially compensating for tissue damage (27).

### Lateralization of Working Memory Functions

Interestingly, we also saw that the associations discussed above between (1) verbal memory and hippocampal volume and (2) visuo-spatial memory and hippocampal functional SPL, were, respectively, driven by (1) the left and (2) the right hippocampus. This is in line with the commonly accepted idea that verbal working memory is left-lateralized while visual working memory is right lateralized [see (38) for a review].

### Healthy Controls Preserved Cognitive Performances and Hippocampal Networks

Healthy controls did not show any significant alterations in memory performances nor in hippocampal MRI metrics over time. This gives us confidence on the robustness of our dataset and allows us to safely interpret alterations seen in patients as consequences of MS.

### Strengths and Limitations

#### Strengths

Strengths of this study include its longitudinal nature over 5 years in a homogeneous population of patients at the early stages of the disease. Additionally, our setting included healthy controls who came back for a 5-year follow-up; a very important advantage since it allows to account for test-retest biases on cognitive tests. Moreover, healthy controls showed no significant evolution in any of the MRI metrics over time, supporting the idea that we can rely on the results observed on our patient's population and attribute them to the evolution of the disease. Altogether, this study gives strong arguments in favor of functional compensation, with regards to the conflicting results around this question (cf. Introduction).

#### Limitations

However, there are some methodological limitations to be considered. First, the number of recruited patients followed over the 5 years is limited by missing data which inherently limits the statistical power. In addition, the number of healthy controls who came back longitudinally is low, raising the concern of the robustness of our control group. Nevertheless, as mentioned before, no significant alteration of MRI metrics was observed in controls over time, suggesting that the study design was performant enough to allow stable comparisons. Also, we would like to highlight the fact that it is very rare to have longitudinal data on a control group, especially over a 5-year period, and that still constitutes a great asset of this study.

Second, the ability of tractography algorithms to detect fibers can be affected by the presence of white matter lesions. Nevertheless, a recent study reported that, even though MS lesions impact tractography algorithms, fiber tracking is still possible and anatomically accurate (39). Second, our DTI data were characterized by only 21 non-collinear directions, which could have an effect on our tractography estimations.

In addition, the parcellation used in this study considers the hippocampus as a whole and do not allow the detection of hippocampal sub-fields. This could be a limitation since different memory subtypes might rely on different hippocampal sub-fields. Moreover, we limited our analysis to the hippocampus, while other regions play important roles in verbal and visuospatial memory—such as the right and left medial temporal cortex (40). Lastly, in this study we limited our investigations to four commonly used metrics from graph theory for the evaluation of hippocampal structural and functional connectivity in order to avoid inflation of type I error due to limited sample size; however, other graph measures, such as modularity, could be analyzed in future studies to provide additional insights.

### Conclusion

In conclusion, our study demonstrated an important interplay between hippocampal-related structural and functional networks in explaining cognitive performances in the early stages of MS. As the structural damage increases, verbal memory performances decrease while functional reorganization seems to be able to maintain visuo-spatial memory performances with strengthened short-distance connections. Considering those results, a future line of study would be to investigate how such functional reorganization can be stimulated in order to delay the appearance of cognitive impairment.

## Data Availability Statement

The raw data supporting the conclusions of this article will be made available by the authors, without undue reservation.

## Ethics Statement

The studies involving human participants were reviewed and approved by Bordeaux University Hospital Ethics Committee. The patients/participants provided their written informed consent to participate in this study.

## Author Contributions

AR, MD, BB, TT, and IK: study concept and design. JB, AR, MD, BB, TT, and IK: analysis and interpretation of the data. JB, TT, and IK: statistical analysis and drafting the manuscript. All authors: revised the manuscript for important intellectual content, contributed to the article, and approved the submitted version.

## Conflict of Interest

The authors declare that this study received funding from Teva Pharmaceuticals Ltd and Merck Serono. The funders were not involved in the study design, collection, analysis, interpretation of data, the writing of this article or the decision to submit it for publication.
