# Supplementary material for: Insights on the Relationship Between Hippocampal Connectivity and Memory Performances at the Early Stage of Multiple Sclerosis
Source: Front Neurol. 2021 May 19;12:667531. doi: 10.3389/fneur.2021.667531 (PMC8170471; doi:10.3389/fneur.2021.667531)

Supplementary Material

**Supplementary Table 1.**

Table S1: Raw scores to the cognitive tests at baseline, year 1 and year 5.

| **Cognitive test** | **Baseline** | | **1 year** | | **5 years** | |
| --- | --- | --- | --- | --- | --- | --- |
|  | **Patients** | **Controls** | **Patients** | **Controls** | **Patients** | **Controls** |
| **SRT-LTS** | 59.594 (8.210) | 60 (10.832) | 60.094 (9.074) | 62 (7.067) | 61.065 (9.313) | 64 (9.191) |
| **SRT-CLTR** | 53.031 (13.015) | 53 (15.556) | 53.969 (12.212) | 54 (14.621) | 56.065 (13.706) | 62 (11.549) |
| **SRT-DR** | 11.188 (1.693) | 11 (1.229) | 11.031 (1.231) | 11 (0.966) | 11.226 (1.309) | 12 (0.948) |
| **BVMTR-learning** | 27.594 (5.797) | 30 (6.074) | 30.156 (4.608) | 30 (5.461) | 29.097 (5.394) | 30 (4.575) |
| **BVMTR-DR** | 10.219 (1.996) | 11 (1.686) | 10.969 (1.636) | 11 (1.646) | 11 (1.461) | 11 (1.154) |

*The data are provided as mean (standard deviation) at each time point. SRT=Selective Reminding Test; LTS=long-term storage; CLTR=consistent long-term retrieval; SRT-DR=delayed recall; BVMTR= Brief Visual Memory Test Revised; BVMTR-DR=delayed recall.*

**Supplementary Figure 1.**

Figure S1: Structural connectome matrices for patients at baseline, year 1 and year 5.

**
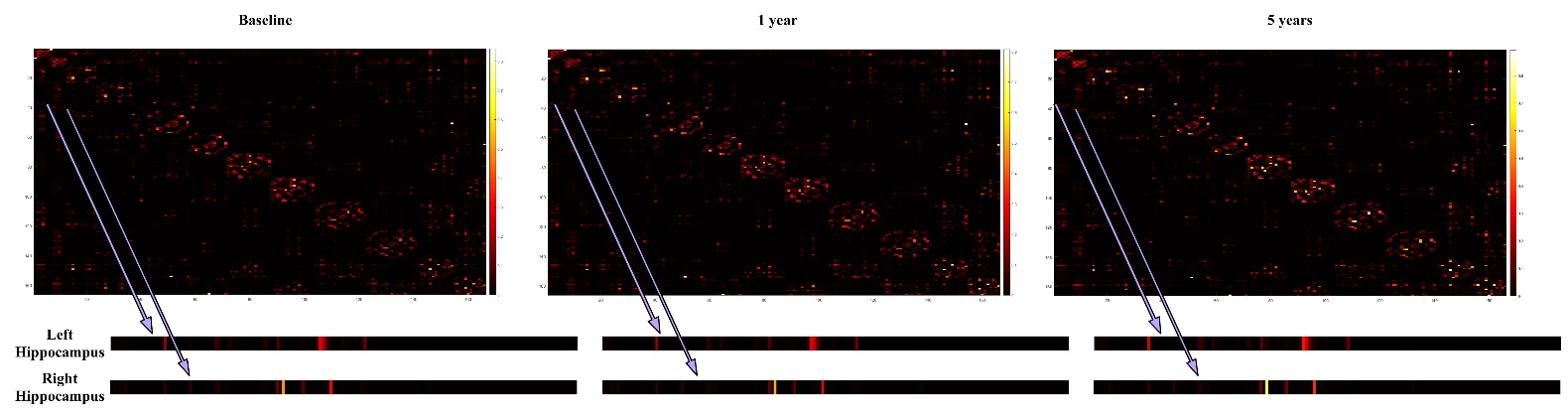
**

**Supplementary Figure 2.**

Figure S2: Functional connectome matrices for patients at baseline, year 1 and year 5.


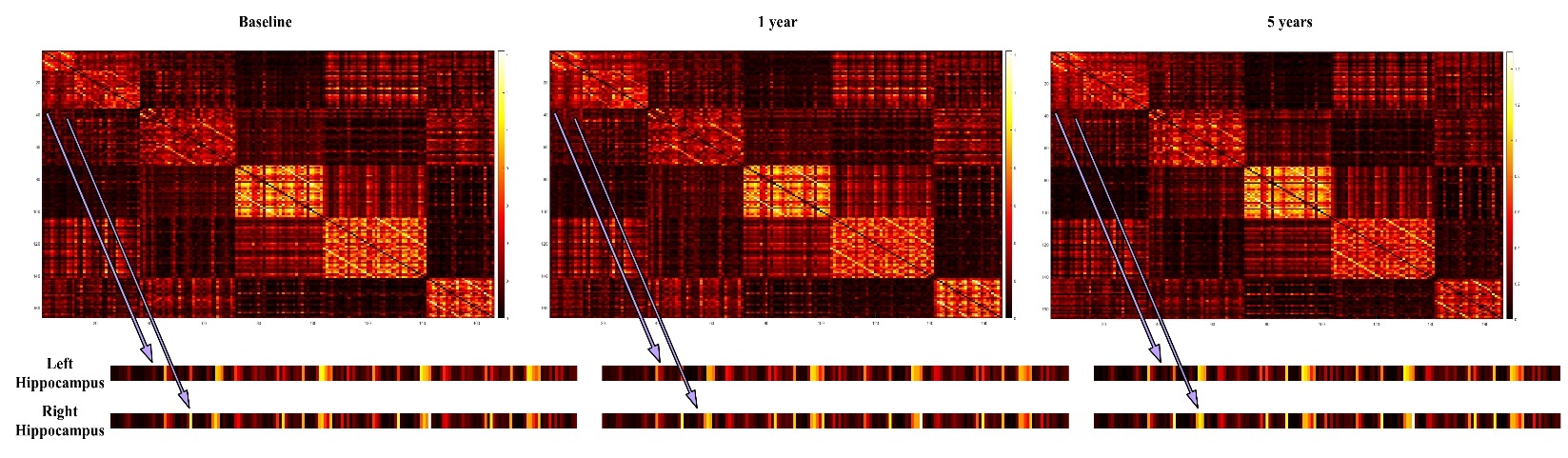

Supplement: Supplementary file 1 [file Data_Sheet_1.docx]
